# Early GH therapy and neurodevelopmental outcome in a child with compound heterozygous *IGF1R* variants

**DOI:** 10.1210/jcemcr/luag081

**Published:** 2026-04-21

**Authors:** Mariana Sá Pinto, Mariana Oliveira, Tomás Ferrão, Fabiana Ramos, Rita Cardoso, Ana Luísa Leite

**Affiliations:** Serviço de Pediatria e Neonatologia da Unidade Local de Saúde Gaia e Espinho, Vila Nova de Gaia 4434-502, Portugal; Serviço de Pediatria e Neonatologia da Unidade Local de Saúde Gaia e Espinho, Vila Nova de Gaia 4434-502, Portugal; Serviço de Pediatria da Unidade Local de Saúde Baixo Vouga, Aveiro 3814-501, Portugal; Serviço de Genética Médica, Hospital Pediátrico de Coimbra, Unidade Local de Saúde de Coimbra, Coimbra 3000-062, Portugal; Unidade de Endocrinologia Pediátrica e Diabetes, Hospital Pediátrico de Coimbra, Unidade Local de Saúde de Coimbra, Coimbra 3000-602, Portugal; Serviço de Pediatria e Neonatologia da Unidade Local de Saúde Gaia e Espinho, Vila Nova de Gaia 4434-502, Portugal; Unidade de Endocrinologia e Diabetologia Pediátrica, Unidade Local de Saúde Gaia e Espinho, Vila Nova de Gaia 4434-502, Portugal

**Keywords:** insulin-like growth factor 1 receptor variants, growth hormone, fetal growth restriction, microcephaly, neurodevelopment

## Abstract

Causal variants in the IGF 1 receptor (*IGF1R*) gene are associated with variable degrees of growth and neurodevelopmental impairment. While heterozygous variants often manifest as less severe phenotypes, homozygous loss-of-function mutations are widely regarded as incompatible with life. Biallelic hypomorphic variants are exceptionally rare and their clinical spectrum remains poorly defined. We report the case of a girl born at term after severe symmetric fetal growth restriction (FGR), identified prenatally through trio whole-exome sequencing as carrying compound heterozygous *IGF1R* variants—c.155G>C (p.Cys52Ser) and c.3476A>G (p.Asp1159Gly). Postnatally, she exhibited microcephaly, dysmorphic features, and marked growth failure. Growth hormone therapy was initiated at 13 months of age, leading to gradual and sustained improvement in head circumference, linear growth, and psychomotor development. We report a rare case of a prenatal diagnosis of compound heterozygous *IGF1R* variants with early GH treatment. The case broadens current knowledge on *IGF1R*-related disorders, shows that survival is possible in compound heterozygous states, and suggests a potential benefit of early GH therapy.

## Introduction

The IGF 1 receptor (IGF1R) has been identified as playing a critical role in somatic growth and neurodevelopment [1 , 2]. The presence of pathogenic variants in *IGF1R* gene has been demonstrated to be associated with several congenital anomalies, including prenatal and postnatal growth restriction, microcephaly, and variable dysmorphic features [1, 2]. Heterozygous variants generally manifest with less severe phenotypes, whereas complete homozygous loss of function is considered incompatible with life [1, 3].

Compound heterozygous variants are exceptionally rare, with only a handful of cases reported to date [1, 2, 4]. The role of GH therapy in these patients remains poorly defined, particularly regarding neurodevelopmental outcomes [2, 5]. This report refers to a child diagnosed prenatally with compound heterozygous variants in the *IGF1R* gene, who not only survived but also received early GH treatment, providing novel insights into the therapeutic potential this intervention.

## Case presentation

A female patient was conceived by in vitro fertilization, in the context of the mother's sixth pregnancy after recurrent miscarriages. Severe symmetric fetal growth restriction (FGR) was detected at 22 weeks of gestation. Amniocentesis with trio whole-exome sequencing identified 2 rare *IGF1R* variants in compound heterozygosity: c.155G>C (p.Cys52Ser) and c.3476A>G (p.Asp1159Gly), consistent with autosomal recessive inheritance, classified as likely pathogenic according to The American College of Medical Genetics and Genomics criteria [6]. Serological analysis for congenital infections was negative. Following genetic counseling, the parents—both confirmed heterozygous carriers without growth failure—decided to proceed with the pregnancy.

The infant was delivered at 37 weeks by cesarean section (pelvic presentation). Apgar scores at the first, fifth, and 10th minutes were respectively 8/9/10. Birth measures confirmed severe growth symmetrical restriction: weight 1485 g (-4.5 SD score [SDS]), length 36.5 cm (-6.8 SDS), and head circumference 28 cm (-5.0 SDS). The infant required continuous positive airway pressure for 72 hours, was treated for hypoglycemia on day 1 (34 mg/dL [SI: 1.9 mmol/L] reference range 30-60 mg/dL [SI: 1.7-3.3 mmol/L]), needed parenteral nutrition until day 3, and had neonatal jaundice (phototherapy). Hospitalization was prolonged for nutritional optimization and weight gain monitoring, and she was discharged at 3 weeks of age.

## Diagnostic assessment

The child was followed in the neonatal clinic and was referred to pediatric endocrinology by the age of 9 months, based on sustained severe growth failure. A thorough examination yielded the following results: weight 5.3 kg (-3.7 SDS), length 59.5 cm (-4.4 SDS) and head circumference 37 cm (-5.1 SDS). Dysmorphic features were identified and included anteverted nares, fifth finger clinodactyly, a sacrococcygeal dimple, delayed dentition, and a systolic cardiac murmur clinically and echocardiographically assessed as functional. Subsequent laboratory investigation revealed serum IGF-1 of 245 ng/mL [SI: 32 nmol/L] (+2.71 SDS for age and sex measured by a chemiluminescent enzyme immunoassay; reference range 8-172 ng/mL [SI: 1.0-22.5 nmol/L]) and IGF binding protein 3 (IGFBP3) of 6.07 mg/L (+0.8 SDS for age and sex measured by a chemiluminescent enzyme immunoassay; reference range 1.5-4.5 mg/L). The remaining evaluation, including ACTH, cortisol, and thyroid function, was normal.

## Treatment

Growth hormone therapy (somatropin, 0.035 mg/kg/day) was initiated by 13 months of age. Although GH is not formally approved for this indication or at this age, retrospective studies in small for gestational age (SGA) children with heterozygous *IGF1R* defects have reported positive and safe responses. Dosage was adjusted according to growth, reaching 0.044 mg/kg/day by the age of 2 years. At that time, approximately 1 year after treatment initiation, serum IGF-1 was 409 ng/mL [SI: 53.6 nmol/L] (+3.79 SDS for age and sex measured by the same chemiluminescent enzyme immunoassay), whereas the remaining endocrine evaluation was normal.

## Outcome and follow-up

This child was also evaluated by the neurodevelopmental clinic, and developmental milestones were progressively achieved, including rolling, supported sitting, and object transfer. A standardized baseline neurodevelopmental assessment using the Schedule of Growing Skills II [7] was performed at 6 months of age, demonstrating overall developmental functioning at approximately the 3-month level. A formal neurodevelopmental reassessment was performed at 18 months, and she demonstrated the ability to walk independently, exhibited a refined pincer grasp, articulated brief sentences, and engaged in social interactions with her peers. The attendance of the child at the daycare facility was successful. Because of evident microcephaly, a neurological detailed examination was conducted and revealed no focal deficits, with all cranial nerves and reflexes appearing to be intact. Neurodevelopmental follow-up showed gradual acquisition of milestones. A repeated assessment using the Schedule of Growing Skills evaluation was conducted at 30 months and her locomotor skills reached age-appropriate levels; cognitive and social domains approximated those of a 24-month child, with only mild residual language delay. A detailed developmental assessment at 30 months confirmed progress across domains, consistent with overall functioning closer to age norm than initially anticipated.

**Figure 1:**
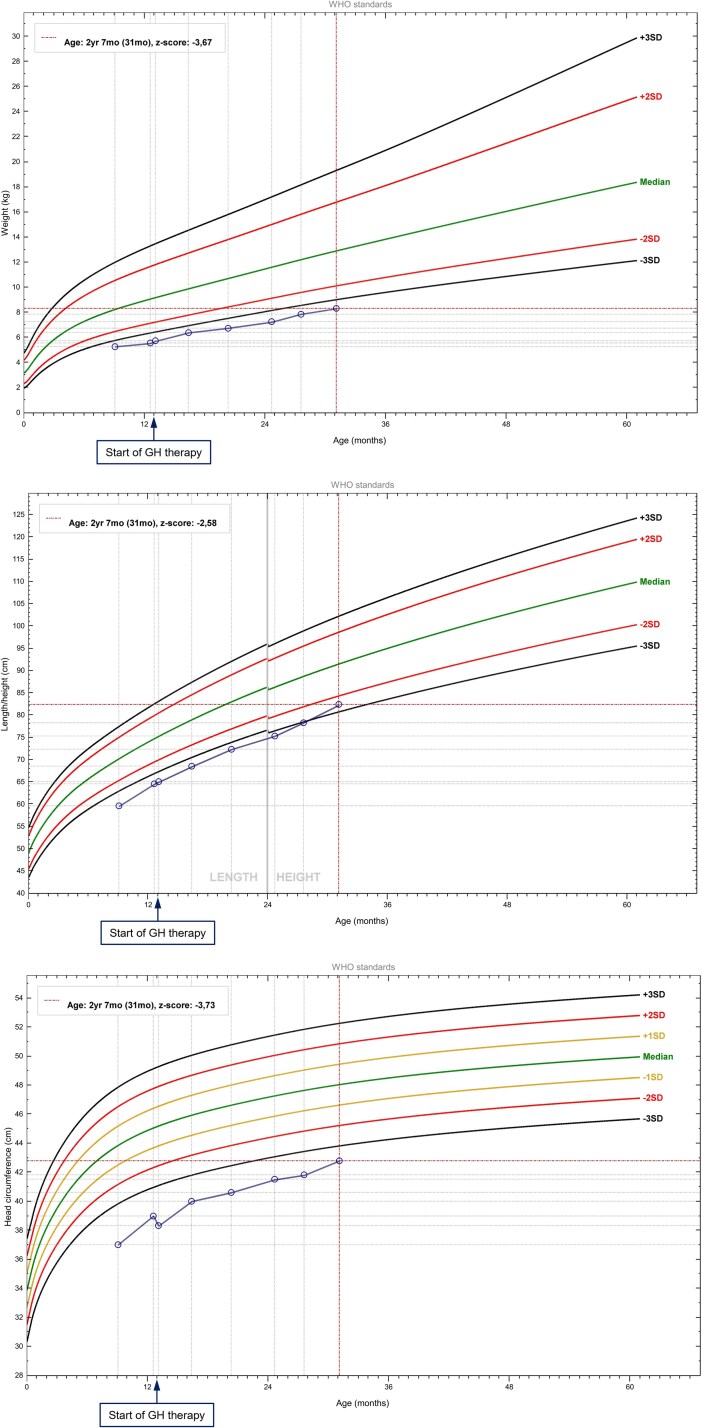
Longitudinal anthropometric SDS evolution—weight, length and head circumference. The arrow marks the start of GH therapy.

Growth monitoring exhibited gradual improvement. At 16 months of age, her weight was 6.38 kg (−3.69 SDS), length 68.5 cm (−3.75 SDS), and head circumference 40 cm (−4.32 SDS). At 24 months, measurements were 7.2 kg (−3.95 SDS), 76 cm (−3.17 SDS), and 41 cm (−4.14 SDS), respectively. At 29 months, growth remained impaired, with a weight of 7.8 kg (−3.6 SDS), length of 80.3 cm (−2.86 SDS), and head circumference of 41.8 cm (−3.7 SDS). By 31 months, her weight had increased to 8.3 kg (−3.67 SDS) and length to 83 cm (−2.39 SDS). Anthropometric SDS were calculated using the World Health Organization Anthro software; this evolution is illustrated in Fig. 1.

## Discussion

This case contributes to improve the scarce literature of children with compound heterozygous variants in the *IGF1R* gene who survive beyond neonatal and early infancy. These cases seem exceedingly uncommon [1]. Large cohort studies using multigene panels or whole-exome sequencing in children with short stature show that monogenic causes account for approximately 10% to 20% of cases. Across these cohorts and published series, *IGF1R* defects are uncommon and predominantly monoallelic, typically involving deletions or missense variants, underscoring the exceptional rarity of biallelic hypomorphic variants [2, 3, 5]. Fang et al described one of the earliest patients with severe FGR and postnatal growth failure resulting from compound heterozygous variants in the *IGF1R* gene [1]. Subsequently, in 2019, Walenkamp et al emphasized that the majority of reported patients with *IGF1R*-related short stature is 1 allele heterozygous, with only 3 compound heterozygous individuals identified [3]. More recently, Çelik et al reported long-term GH therapy in a patient with an *IGF1R* deletion, started by the age of 12 years, adding new evidence to the potential benefit of GH in these patients [4]. Ocaranza et al confirmed that since the first descriptions in 2003, only a few patients with this genetic pattern have been documented [8]. In cohorts of SGA children treated with GH, retrospective genetic evaluations have sometimes revealed *IGF1R* variants in nonresponders or partial responders, highlighting the potential underdiagnosis of receptor-level defects [9].

This case is different in 2 key aspects. First, the diagnosis was established in utero, thus enabling early genetic counseling and personalized monitoring. Second, and most significantly, to our knowledge, this is one of the first cases with biallelic variants in the *IGF1R* gene to receive GH therapy early in life.

Somatropin exerts both direct effects via the GH receptor and indirect effects through IGF-1, which promotes growth-plate cartilage proliferation and hypertrophy. In children born SGA without adequate catch-up growth, GH therapy has been established as a treatment for more than 2 decades, with substantial real-world evidence of its effectiveness [3, 4, 8]. Large registries and cohort studies (including Kabi International Growth Study, which enrolled more than 80 000 children, ∼10% SGA) have documented sustained height gains and acceptable long-term safety. However, in the context of *IGF1R* defects, treatment response is heterogeneous and often partial and requires individualized decision-making and careful longitudinal monitoring [3]. Furthermore, several studies, as well as a recent international consensus guideline, have reported modest but measurable benefits in cognitive and psychosocial domains among GH-treated SGA children [10, 11]. Some trials have even linked neurocognitive improvement to parallel increases in head circumference during treatment [10, 12-15].

In this patient, despite severe prenatal growth restriction and extreme microcephaly, neurodevelopmental progression was observed, with steady acquisition of milestones and clear functional improvement over time. The progressive, albeit gradual, increase in head growth occurred in parallel with developmental advancements. Although causality cannot be inferred from a single uncontrolled observation, experimental and translational studies have independently demonstrated a critical role of the GH and IGF-1 axis in brain development and function [16].

Published data on compound heterozygous *IGF1R* variants are scarce and often outdated. The paucity of cases and limited longitudinal follow-up restrict our ability to generalize prognoses or treatment strategies. The literature on biallelic *IGF1R* defects is extremely limited. Many affected pregnancies are medically interrupted or result in miscarriage, making liveborn follow-up rare. The liveborn survivors described rely mostly on older case series, underscoring the value of this new report.

## Learning points

- Early recognition of *IGF1R*-related growth disorders enables personalized monitoring and timely initiation of therapy.
- Early initiation of GH therapy, including during infancy, appears to be important for optimizing growth trajectories and may be associated with improved neurodevelopmental outcomes in selected cases.
- This case expands the phenotypic spectrum of *IGF1R* defects and illustrates the use of GH therapy in a severe biallelic presentation, underscoring the need for individualized management and longitudinal follow-up.

## Contributors

All authors made individual contributions to authorship. M.S.P. was responsible for data collection and clinical data analysis. M.O. and T.F. contributed to clinical data analysis. F.R. participated in genetic data interpretation and contributed to the diagnostic evaluation. R.C. contributed to the initial clinical assessment and review of the diagnostic work-up. A.L.L. provided clinical supervision and contributed to the treatment strategy and follow-up of the patient. All authors reviewed and approved the final version of the manuscript.

## Data Availability

Data sharing is not applicable to this article as no datasets were generated or analyzed during this study.
